# Type III Secretion in *Yersinia*: Injectisome or Not?

**DOI:** 10.1371/journal.ppat.1002669

**Published:** 2012-05-10

**Authors:** Tomas Edgren, Åke Forsberg, Roland Rosqvist, Hans Wolf-Watz

**Affiliations:** 1 Department of Molecular Biology, Laboratory for Molecular Infection Medicine Sweden (MIMS), Umeå Centre for Microbial Research (UCMR), Umeå University, Umeå, Sweden; 2 The Electron Microscopy Platform at KBC, Umeå University, Umeå, Sweden; University of North Carolina at Chapel Hill, United States of America

## Introduction

Under certain conditions, pathogens like *Yersinia* secrete high levels of proteins. This ability correlates with cytotoxicity and virulence. The complete inactivity of the secreted proteins when added directly to host cells stimulated research to disclose this “non-AB toxin mechanism” of virulence, and eventually led to the discovery of the type III secretion mechanism and the “injection model” for virulence effector targeting. Recent studies question this model and suggest that the T3SS effector-targeting mechanism may resemble the classical AB toxin delivery mechanism.

## The Ca^2+^ Paradox

Human pathogenic *Yersinia* species are uniquely dependent on millimolar concentrations of Ca^2+^ when grown at 37°C. This phenotype is characterized by normal growth at 37°C in the presence of Ca^2+^, and growth restriction in Ca^2+^-depleted medium [1]. This unusual Ca^2+^ requirement has been linked to the presence of a virulence plasmid [2], originally discovered by Zink et al. [3]. The plasmid encoded a set of proteins (*Yersinia* outer proteins, or Yops) [4] that were secreted into the culture supernatant in massive amounts during growth restriction in the absence of Ca^2+^ [5], [6]. The coupling of the Yops to the virulence plasmid indicated strongly that they were essential virulence determinants. This hypothesis was further supported when convalescence sera obtained from *Yersinia*-infected patients were shown to contain antibodies recognizing the secreted Yop proteins [4], leading to the hypothesis that low intracellular Ca^2+^ inside mammalian cells induced expression of the Yop virulon during infection. The role of calcium was thus paradoxical as conditions promoting Yop secretion resulted in growth restriction.

## Polarized Translocation of Yop Effectors

Cavanaugh and Randall were the first to demonstrate that blockage of phagocytosis is crucial for *Yersinia* to cause disease [7]. The significance of this finding was later strengthened by the finding that the bacteria replicate predominantly at extracellular sites during infection [8]. YopH and YopE were essential to the ability to block phagocytosis [9]. These data demonstrated that *Yersinia* is an extracellular pathogen and that Yop expression is a prerequisite for virulence, and also raised the question of how the pathogen was able to affect target cells from its extracellular location—an especially intriguing question because, unlike AB toxins, the addition of purified Yops to cultured cells did not cause any obvious effects.

The first important discovery to resolve this issue was the finding that microinjection of purified YopE into target cells induced a cytotoxic response [10], demonstrating that YopE must be translocated into target cells to elicit its biological function. It was also concluded that in addition to YopE, the secreted protein YopD was essential for the delivery of YopE across the target cell membrane by infecting bacteria [10]. Confocal imaging and biochemical fractionation were used to demonstrate that contact between *Yersinia* and the target cell induces Yop expression accompanied by the translocation of YopE into target cells [11], [12]. The majority of YopE was present in the target cell cytosol and no YopE was detected in the culture medium, indicating that Yop translocation was polarized and occurred only at the zone of contact between the bacterium and the target cell [12]. Translocation of Yop effectors into target cells was later demonstrated by another approach using an elegant reporter system [13].

## Does the Type III Secretion System Function as a Microsyringe?

Cornelis and co-workers were the first to demonstrate that the virulence plasmid of pathogenic *Yersinia* encodes a dedicated Yop secretion system [14]. Some genes identified by Cornelis et al. had homologous counterparts in plant pathogens, and Reeves and Salmond realized that these different pathogens exhibited a common secretion pathway, dubbed “Type III secretion” (T3S) [15]. An additional set of *Yersinia* genes was identified that displayed remarkable homology to corresponding genes in *Salmonella* and *Shigella* [16]. The idea that highly conserved secretory systems could allow heterologous secretion was based on these observations, and corroborated by experimental evidence demonstrating that *S. typhimurium* secreted YopE via a T3SS-dependent mechanism. Both secretion and cell contact-dependent translocation of effectors into the target cells were functionally conserved among *yersiniae*, *salmonellae*, and *shigellae* [17], which led us to suggest that the T3SS-dependent secretion and translocation of effector proteins were highly conserved among different pathogens and occurred through a “microinjection mechanism.”

That the T3SSs are organized into supra-molecular structures known as needle complexes was first shown in *S. typhimurium* [18]. This structure, which is believed to be common to all T3SSs, resembles a syringe with a base structure and a needle-like protrusion extending from the surface of the pathogen. The entire needle complex is traversed by a fine, hollow tube that may allow passage of effector proteins across the bacterial cell envelope [19]. Due to the suggestive similarities between the needle complex and a syringe, it has been generally accepted that the effectors travel directly through the needle complex from the bacterial cytosol into the lumen of the eukaryotic target cell. However, no experimental results have been presented to demonstrate that the effectors are secreted through the needle structure.

## Are the Effectors Translocated through a Pore Formed in the Host Cell Membrane?

The secreted substrates can be divided into two functional classes: effectors are delivered into the target cell where they elicit a biological response, while translocators facilitate the delivery of effectors across the plasma membrane [10], [12], [13]. A key signature of translocators is the putative membrane spanning domain(s), which has some similarities to pore-forming toxins [20]. The first translocator protein found to exhibit pore-forming and hemolytic activities was *Shigella* IpaB [21]. Subsequently, the *Yersinia* homologue YopB was found to be essential for Yop effector translocation; similar to IpaB, YopB also induces erythrocyte hemolysis [22], [23]. The pore-forming ability of YopB correlates with functional translocation, supporting the idea that the effectors are delivered through a pore in the host cell membrane. Recent work has revealed that LcrV (initially thought to be a translocator protein) is localized at the tip of the needle complex [24]. LcrV has also been proposed to be essential for insertion of the hydrophobic translocators YopB and YopD into target cell membranes. These properties of LcrV agree nicely with the “injection model.”

However, all findings are not compatible with this model. For example, Sasakawa and co-workers reported that the *Shigella* translocators IpaB, IpaC, and IpaD are surface-localized before target cell contact [25]. The majority of these proteins are rapidly released after target cell contact is established, indicating that *Shigella* senses target cell contact and responds accordingly. Furthermore, latex beads coated with purified Ipa protein complexes are internalized by target cells through a mechanism that resembles the active, T3SS-dependent engulfment of *Shigella* [26]. The apparent surface localization of these proteins before target cell contact is difficult to reconcile with the injection model. On the other hand, recent studies in *Shigella* demonstrating that IpaB and subsequently IpaC are recruited to the tip of the needle from its location at the bacterial surface in response to target cell contact lend support to the injection model [27]. Nevertheless, these results do not support the idea that the T3SS forms a conduit that allows both translocators and effectors to be secreted in one step by the same T3SS, since at least the translocators are secreted to the surface before eukaryotic cell contact has been established.

## Binary AB Toxin Revisited?

**Figure 1:**
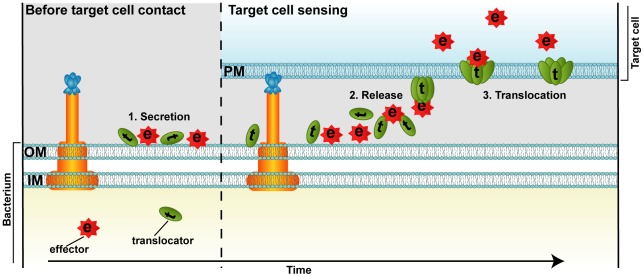
Proposed model for T3SS-dependent protein translocation by a binary AB toxin like mechanism. T3SS translocators (t) and effectors (e) are secreted by the T3SS across the bacterial envelope (IM and OM) to the surface of the cell before host cell contact (1). Target cell sensing results in release of the surface localized T3SS substrates (2). The translocators (t) assemble into a pore in the target cell plasma membrane (PM) and mediate the translocation of the effectors (e) into the target cell cytoplasm (3).

Recent attempts to visualize T3SS substrates during infection using immunogold labeling and transmission electron microscopic analysis revealed that the majority of Yop translocators and effectors are present on the surface of the bacterium before target cell contact [28]. This finding was surprising because the injection model predicts that the T3SS substrates should be present in a secretion competent conformation in the bacterial cytosol before target cell contact. Remarkably, a *yopH* null mutant coated with purified YopH was translocation competent and complemented the mutant phenotype. The translocation of surface-localized YopH and YopH-β-lactamase reporters is also T3SS dependent and requires functional translocators, as well as a specific translocation domain present in YopH. The N-terminal secretion signal is redundant for translocation of externally added YopH, demonstrating that secretion and translocation are two separate events. Importantly, T3SS-dependent translocation of externally added YopH has also been achieved in *S. typhimurium*, indicating that the ability to translocate surface-localized effectors is conserved among different T3SS-dependent pathogens. Taken together, these data support a two-step model in which the T3SS substrates are secreted first. After target cell sensing, these surface-localized effectors are translocated across the target cell plasma membrane (Figure 1). The translocation step could superficially be compared to the delivery of binary AB toxins: the hydrophobic T3SS translocators resemble the pore-forming B-moiety that mediates the translocation of the catalytic A-moiety (Yop effectors). In this scenario, tight binding of the bacterium to the target cell provides the micro-environment required for efficient translocation of effectors across the plasma membrane. This requires that the surface-localized T3SS substrates are released when the bacterium senses target cell contact, leading to derepression of the *yop* regulon via the T3SS [11]. Because increased *yop* expression is coupled to increased Yop translocation, release may be coupled to the needle complex. Therefore, we suggest a sensory role for the needle complex, thereby explaining the requirement for target cell contact in T3SS-dependent effector delivery. However, our results do not exclude the injection model, because it is still possible for the two processes to operate in parallel. Obviously, more work is needed before the molecular details of the translocation are resolved.
